# Evidence-based models of care for the treatment of alcohol use disorder in primary health care settings: protocol for systematic review

**DOI:** 10.1186/s13643-019-1157-7

**Published:** 2019-11-13

**Authors:** Susan A. Rombouts, James Conigrave, Eva Louie, Paul Haber, Kirsten C. Morley

**Affiliations:** 1 Discipline of Addiction Medicine, Central Clinical School, Faculty of Medicine and Health, University of Sydney, Sydney, NSW Australia; 2 NHMRC Centre of Research Excellence in Indigenous Health and Alcohol, Central Clinical School, Faculty of Medicine and Health, University of Sydney, Sydney, NSW Australia; 3 Drug Health Services, Royal Prince Alfred Hospital, Camperdown, NSW Australia

**Keywords:** Model of care, Alcohol, Addiction, Primary health care, Systematic review

## Abstract

**Background:**

Alcohol use disorder (AUD) is highly prevalent and accounts globally for 1.6% of disability-adjusted life years (DALYs) among females and 6.0% of DALYs among males. Effective treatments for AUDs are available but are not commonly practiced in primary health care. Furthermore, referral to specialized care is often not successful and patients that do seek treatment are likely to have developed more severe dependence. A more cost-efficient health care model is to treat less severe AUD in a primary care setting before the onset of greater dependence severity. Few models of care for the management of AUD in primary health care have been developed and with limited implementation. This proposed systematic review will synthesize and evaluate differential models of care for the management of AUD in primary health care settings.

**Methods:**

We will conduct a systematic review to synthesize studies that evaluate the effectiveness of models of care in the treatment of AUD in primary health care. A comprehensive search approach will be conducted using the following databases; MEDLINE (1946 to present), PsycINFO (1806 to present), Cochrane Database of Systematic Reviews, Cochrane Central Register of Controlled Trials (CENTRAL) (1991 to present), and Embase (1947 to present).

Reference searches of relevant reviews and articles will be conducted. Similarly, a gray literature search will be done with the help of Google and the gray matter tool which is a checklist of health-related sites organized by topic. Two researchers will independently review all titles and abstracts followed by full-text review for inclusion. The planned method of extracting data from articles and the critical appraisal will also be done in duplicate. For the critical appraisal, the Cochrane risk of bias tool 2.0 will be used.

**Discussion:**

This systematic review and meta-analysis aims to guide improvement of design and implementation of evidence-based models of care for the treatment of alcohol use disorder in primary health care settings. The evidence will define which models are most promising and will guide further research.

**Protocol registration number:**

PROSPERO CRD42019120293.

## Background

It is well recognized that alcohol use disorders (AUD) have a damaging impact on the health of the population. According to the World Health Organization (WHO), 5.3% of all global deaths were attributable to alcohol consumption in 2016 [1]. The 2016 Global Burden of Disease Study reported that alcohol use led to 1.6% (95% uncertainty interval [UI] 1.4–2.0) of total DALYs globally among females and 6.0% (5.4–6.7) among males, resulting in alcohol use being the seventh leading risk factor for both premature death and disability-adjusted life years (DALYs) [2]. Among people aged 15–49 years, alcohol use was the leading risk factor for mortality and disability with 8.9% (95% UI 7.8–9.9) of all attributable DALYs for men and 2.3% (2.0–2.6) for women [2]. AUD has been linked to many physical and mental health complications, such as coronary heart disease, liver cirrhosis, a variety of cancers, depression, anxiety, and dementia [2, 3]. Despite the high morbidity and mortality rate associated with hazardous alcohol use, the global prevalence of alcohol use disorders among persons aged above 15 years in 2016 was stated to be 5.1% (2.5% considered as harmful use and 2.6% as severe AUD), with the highest prevalence in the European and American region (8.8% and 8.2%, respectively) [1].

Effective and safe treatment for AUD is available through psychosocial and/or pharmacological interventions yet is not often received and is not commonly practiced in primary health care. While a recent European study reported 8.7% prevalence of alcohol dependence in primary health care populations [4], the vast majority of patients do not receive the professional treatment needed, with only 1 in 5 patients with alcohol dependence receiving any formal treatment [4]. In Australia, it is estimated that only 3% of individuals with AUD receive approved pharmacotherapy for the disorder [5, 6]. Recognition of AUD in general practice uncommonly leads to treatment before severe medical and social disintegration [7]. Referral to specialized care is often not successful, and those patients that do seek treatment are likely to have more severe dependence with higher levels of alcohol use and concurrent mental and physical comorbidity [4].

Identifying and treating early stage AUDs in primary care settings can prevent condition worsening. This may reduce the need for more complex and more expensive specialized care. The high prevalence of AUD in primary health care and the chronic relapsing character of AUD make primary care a suitable and important location for implementing evidence-based interventions. Successful implementation of treatment models requires overcoming multiple barriers. Qualitative studies have identified several of those barriers such as limited time, limited organizational capacity, fear of losing patients, and physicians feeling incompetent in treating AUD [8–10]. Additionally, a recent systematic review revealed that diagnostic sensitivity of primary care physicians in the identification of AUD was 41.7% and that only in 27.3% alcohol problems were recorded correctly in primary care records [11].

Several models for primary care have been created to increase identification and treatment of patients with AUD. Of those, the model, screening, brief interventions, and referral to specialized treatment for people with severe AUD (SBIRT [12]) is most well-known. Multiple systematic reviews exist, confirming its effectiveness [13–15], although implementation in primary care has been inadequate. Moreover, most studies have looked primarily at SBIRT for the treatment of less severe AUD [16]. In the treatment of severe AUD, efficacy of SBIRT is limited [16]. Additionally, many patient referred to specialized care often do not attend as they encounter numerous difficulties in health care systems including stigmatization, costs, lack of information about existing treatments, and lack of non-abstinence-treatment goals [7]. An effective model of care for improved management of AUD that can be efficiently implemented in primary care settings is required.

### Review objective

This proposed systematic review will synthesize and evaluate differential models of care for the management of AUD in primary health care settings. We aim to evaluate the effectiveness of the models of care in increasing engagement and reducing alcohol consumption.

By providing this overview, we aim to guide improvement of design and implementation of evidence-based models of care for the treatment of alcohol use disorder in primary health care settings.

## Methods

The systematic review is registered in PROSPERO international prospective register of systematic reviews (CRD42019120293) and the current protocol has been written according to the Preferred Reporting Items for Systematic Reviews and Meta-Analyses Protocols (PRISMA-P) recommended for systematic reviews [17]. A PRISMA-P checklist is included as Additional file 1.

### Eligibility criteria

Criteria for considering studies for this review are classified by the following:

#### Study design

Both individualized and cluster randomized trials will be included. Masking of patients and/or physicians is not an inclusion criterion as it is often hard to accomplish in these types of studies.

#### Population

Patients in primary health care who are identified (using screening tools or by primary health care physician) as suffering from AUD (from mild to severe) or hazardous alcohol drinking habits (e.g., comorbidity, concurrent medication use). Eligible patients need to have had formal assessment of AUD with diagnostic tools such as Diagnostic and Statistical Manual of Mental Disorders (DSM-IV/V) or the International Statistical Classification of Diseases and Related Health Problems (ICD-10) and/or formal assessment of hazardous alcohol use assessed by the Comorbidity Alcohol Risk Evaluation Tool (CARET) or the Alcohol Use Disorders Identification test (AUDIT) and/or alcohol use exceeding guideline recommendations to reduce health risks (e.g., US dietary guideline (2015–2020) specifies excessive drinking for women as ≥ 4 standard drinks (SD) on any day and/or ≥ 8 SD per week and for men ≥ 5 SD on any day and/or ≥ 15 SD per week).

Studies evaluating models of care for additional diseases (e.g., other dependencies/mental health) other than AUD are included when they have conducted data analysis on the alcohol use disorder patient data separately or when 80% or more of the included patients have AUD.

#### Intervention

The intervention should consist of a model of care; therefore, it should include multiple components and cover different stages of the care pathway (e.g., identification of patients, training of staff, modifying access to resources, and treatment). An example is the Chronic Care Model (CCM) which is a primary health care model designed for chronic (relapsing) conditions and involves six elements: linkage to community resources, redesign of health care organization, self-management support, delivery system redesign (e.g., use of non-physician personnel), decision support, and the use of clinical information systems [18, 19].

As numerous articles have already assessed the treatment model SBIRT, this model of care will be excluded from our review unless the particular model adds a specific new aspect. Also, the article has to assess the effectiveness of the model rather than assessing the effectiveness of the particular treatment used. Because identification of patients is vital to including them in the trial, a care model that only evaluates either patient identification or treatment without including both will be excluded from this review.

#### Comparator

Model effectiveness may be in comparison with the usual care or a different treatment model.

#### Outcomes

Included studies need to include at least one of the following outcome measures: alcohol consumption, treatment engagement, uptake of pharmacological agents, and/or quality of life.

#### Study design

Solely quantitative research will be included in this systematic review (e.g., randomized controlled trials (RCTs) and cluster RCTs). We will only include peer-reviewed articles.

#### Restrictions (language/time period)

Studies published in English after 1 January 1998 will be included in this systematic review.

#### Setting

Studies have to be conducted in primary health care settings as such treatment facilities need to be physically in or attached to the primary care clinic. Examples are co-located clinics, veteran health primary care clinic, hospital-based primary care clinic, and community primary health clinics. Specialized primary health care clinics such as human immunodeficiency virus (HIV) clinics are excluded from this systematic review. All studies were included, irrespective of country of origin.

### Search strategy and information sources

A comprehensive search will be conducted. The following databases will be consulted: MEDLINE (1946 to present), PsycINFO (1806 to present), Cochrane Database of Systematic Reviews, Cochrane Central Register of Controlled Trials (CENTRAL) (1991 to present), and Embase (1947 to present). Initially, the search terms will be kept broad including alcohol use disorder (+synonyms), primary health care, and treatment to minimize the risk of missing any potentially relevant articles. Depending on the number of references attained by this preliminary search, we will add search terms referring to models such as models of care, integrated models, and stepped-care models, to limit the number of articles. Additionally, we will conduct reference searches of relevant reviews and articles. Similarly, a gray literature search will be done with the help of Google and the Gray Matters tool which is a checklist of health-related sites organized by topic. The tool is produced by the Canadian Agency for Drugs and Technologies in Health (CADTH) [20].

See Additional file 2 for a draft of our search strategy in MEDLINE.

### Data collection

The selection of relevant articles is based on several consecutive steps. All references will be managed using EndNote (EndNote version X9 Clarivate Analytics). Initially, duplicates will be removed from the database after which all the titles will be screened with the purpose of discarding clearly irrelevant articles. The remaining records will be included in an abstract and full-text screen. All steps will be done independently by two researchers. Disagreement will lead to consultation of a third researcher.

### Data extraction and synthesis

Two researchers will extract data from included records. At the conclusion of data extraction, these two researchers will meet with the lead author to resolve any discrepancies.

In order to follow a structured approach, an extraction form will be used. Key elements of the extraction form are information about design of the study (randomized, blinded, control), type of participants (alcohol use, screening tool used, socio-economic status, severity of alcohol use, age, sex, number of participants), study setting (primary health care setting, VA centers, co-located), type of intervention/model of care (separate elements of the models), type of health care worker (primary, secondary (co-located)), duration of follow-up, outcome measures used in the study, and funding sources. We do not anticipate having sufficient studies for a meta-analysis. As such, we plan to perform a narrative synthesis. We will synthesize the findings from the included articles by cohort characteristics, differential aspects of the intervention, controls, and type of outcome measures.

Sensitivity analyses will be conducted when issues suitable for sensitivity analysis are identified during the review process (e.g., major differences in quality of the included articles).

### Potential meta-analysis

In the event that sufficient numbers of effect sizes can be extracted, a meta-analytic synthesis will be performed. We will extract effect sizes from each study accordingly. Two effect sizes will be extracted (and transformed where appropriate). Categorical outcomes will be given in log odds ratios and continuous measures will be converted into standardized mean differences. Variation in effect sizes attributable to real differences (heterogeneity) will be estimated using the inconsistency index (*I*^2^) [21, 22]. We anticipate high degrees of variation among effect sizes, as a result moderation and subgroup-analyses will be employed as appropriate. In particular, moderation analysis will focus on the degree of heterogeneity attributable to differences in cohort population (pre-intervention drinking severity, age, etc.), type of model/intervention, and study quality. We anticipate that each model of care will require a sub-group analysis, in which case a separate meta-analysis will be performed for each type of model. Small study effect will be assessed with funnel plots and Egger’s symmetry tests [23]. When we cannot obtain enough effect sizes for synthesis or when the included studies are too diverse, we will aim to illustrate patterns in the data by graphical display (e.g., bubble plot) [24].

### Critical appraisal of studies

All studies will be critically assessed by two researchers independently using the Revised Cochrane risk-of-bias tool (RoB 2) [25]. This tool facilitates systematic assessment of the quality of the article per outcome according to the five domains: bias due to (1) the randomization process, (2) deviations from intended interventions, (3) missing outcome data, (4) measurement of the outcome, and (5) selection of the reported results. An additional domain 1b must be used when assessing the randomization process for cluster-randomized studies.

Meta-biases such as outcome reporting bias will be evaluated by determining whether the protocol was published before recruitment of patients. Additionally, trial registries will be checked to determine whether the reported outcome measures and statistical methods are similar to the ones described in the registry. The gray literature search will be of assistance when checking for publication bias; however, completely eliminating the presence of publication bias is impossible.

Similar to article selection, any disagreement between the researchers will lead to discussion and consultation of a third researcher. The strength of the evidence will be graded according to the Grading of Recommendations Assessment, Development and Evaluation (GRADE) approach [26].

### Outcomes

The primary outcome measure of this proposed systematic review is the consumption of alcohol at follow-up. Consumption of alcohol is often quantified in drinking quantity (e.g., number of drinks per week), drinking frequency (e.g., percentage of days abstinent), binge frequency (e.g., number of heavy drinking days), and drinking intensity (e.g., number of drinks per drinking day). Additionally, outcomes such as percentage/proportion included patients that are abstinent or considered heavy/risky drinkers at follow-up. We aim to report all these outcomes. The consumption of alcohol is often self-reported by patients. When studies report outcomes at multiple time points, we will consider the longest follow-up of individual studies as a primary outcome measure.

Depending on the included studies, we will also consider secondary outcome measures such as treatment engagement (e.g., number of visits or pharmacotherapy uptake), economic outcome measures, health care utilization, quality of life assessment (physical/mental), alcohol-related problems/harm, and mental health score for depression or anxiety.

## Discussion

This proposed systematic review will synthesize and evaluate differential models of care for the management of AUD in primary health care settings.

Given the complexities of researching models of care in primary care and the paucity of a focus on AUD treatment, there are likely to be only a few studies that sufficiently address the research question. Therefore, we will do a preliminary search without the search terms for model of care. Additionally, the search for online non-academic studies presents a challenge. However, the Gray Matters tool will be of guidance and will limit the possibility of missing useful studies. Further, due to diversity of treatment models, outcome measures, and limitations in research design, it is possible that a meta-analysis for comparative effectiveness may not be appropriate. Moreover, in the absence of large, cluster randomized controlled trials, it will be difficult to distinguish between the effectiveness of the treatment given and that of the model of care and/or implementation procedure. Nonetheless, we will synthesize the literature and provide a critical evaluation of the quality of the evidence.

This review will assist the design and implementation of models of care for the management of AUD in primary care settings. This review will thus improve the management of AUD in primary health care and potentially increase the uptake of evidence-based interventions for AUD.

## Supplementary information

**Additional file 1.** PRISMA-P 2015 Checklist.

**Additional file 2.** Draft search strategy MEDLINE. Search strategy.

## Data Availability

Not applicable.

## Abbreviations

AUD: Alcohol use disorder
AUDIT: Alcohol Use Disorders Identification test
CADTH: Canadian Agency for Drugs and Technologies in Health
CARET: The Comorbidity Alcohol Risk Evaluation
CENTRAL: Cochrane Central Register of Controlled Trials
DSM-IV/V: Diagnostic and Statistical Manual of Mental Disorders
HIV: Human immunodeficiency virus
ICD: 10 - International Statistical Classification of Diseases and Related Health Problems
PRISMA-P: Preferred Reporting Items for Systematic Reviews and Meta-Analyses Protocols
SBIRT: Screening, brief intervention, referral to specialized treatment
SD: Standard drinks
WHO: World Health Organization
