# Supplementary material for: Evidence-based models of care for the treatment of alcohol use disorder in primary health care settings: protocol for systematic review
Source: Syst Rev. 2019 Nov 13;8:275. doi: 10.1186/s13643-019-1157-7 (PMC6852723; doi:10.1186/s13643-019-1157-7)
Supplement: Supplementary file 2 — Additional file 2. Draft search strategy MEDLINE. Search strategy. [file 13643_2019_1157_MOESM2_ESM.docx]

**Additional file 2** *(draft search strategy MEDLINE)*

Search strategy

- Draft of at least one database

| Concept | Description of concept | Research Terms |
| --- | --- | --- |
| A | Primary Health Care (PHC) | exp Primary Health Care/ OR exp General Practice/ OR Primary Care.mp |
| B | Alcohol Use Disorder | exp Alcoholism/ OR  exp Alcohol Drinking/ OR alcohol dependence.mp. OR alcohol problems.mp. OR hazardous drinking.mp. OR problem drinking.mp. OR AUD.mp |
| C | Treatment | exp disease management/ OR treatment.mp. OR intervention.mp. |
| D | Model of care | models of care.mp. OR  exp "Delivery of Health Care, Integrated"/ OR Patient Care Team/ OR shared care.mp OR Collaborative care.mp OR  stepped care.mp. OR  multi-faceted care.mp. OR  Interdisciplinary treatment approach.mp. OR nurse practitioners/ OR exp family nurse practitioners/ OR exp nurse specialists/ OR specialist liaison.mp. OR Chronic Disease/ OR Chronic Care.mp |

- Search: A+B+C+D
